# CD99: A potential Diagnostic Marker for Differentiating Sub-ependymal Giant Cell Astrocytoma From Other Mimickers: A Report of Five Cases

**Published:** 2017-07-01

**Authors:** Alireza Sadeghipour, Navid Abdi, Pegah Babaheidarian

**Affiliations:** 1 *Dept of Pathology, Iran university of medical sciences, Tehran, Iran*

**Keywords:** Sub-ependymal Giant Cell Astrocytoma, CD99, Immunohistochemical Staining, Tumor

## Abstract

**Background:**

Tuberous sclerosis (TSC) is inherited as an autosomal dominant disease, characterized by skin lesion and tubers in vital organs, especially brain in three categories including subependymal nodules, cortical tubers and subependymal giant cell astrocytoma. Subependymal giant cell astrocytoma (SEGA) is an indolent neoplasm which usually arises at the cauda thalamic groove near foramen monro, although it occurs usually in the clinical settings of TSC, a few number of SEGA has been reported without such history. Its morphology with special cytoarchitecture could be mistaken with other glial brain tumors with similar morphology. Therefore, investigating new markers for differentiating SEGA from other mimickers seems logical rather than other glioneural immunohistochemical markers introduced before.

**Case studies:**

We investigated CD99 expression in SEGA as an adjunctive marker for diagnostic purposes. Five reported cases of SEGA were studied and all of them showed CD99 expression besides usual glioneural markers.

**Conclusion:**

CD99 may be a useful adjunctive marker in differentiating SEGA from other mimickers

## Introduction

Tuberous sclerosis is inherited as an autosomal dominant disease caused by mutation of one of two tumor suppressor genes known as TSC1 and TSC2. It is characterized by skin lesion and tubers in vital organs specifically brain in three categories, including subependymal nodules, cortical tubers and subependymal giant cell astrocytoma (1, 2).

Subependymal giant cell astrocytoma (SEGA) is an indolent neoplasm which usually arises at the cauda thalamic groove near foramen monro and classified as grade I glioma by the World Health Organization (WHO). These tumors are clinically suspected when obstructive hydrocephaly, focal neurological deficit and symptoms of elevated intracranial pressure are found in children or adults with a history of tuberous sclerosis (3, 4). These tumors exhibit specific cytoarchitecture with spindle and epitheliod cells admixed with large giant cells with a ganglion like appearance. These histological features may overlap with its main differential diagnosis, gemistocytic astrocytoma. To investigate characteristic of these lesions, multiple immunohistochemical and ultrastructural studies had been performed before, which showed evidence of glioneural differentiation in SEGA (5, 6). 

In the literature, immunoreactivity for product of MIC2 gene known as CD99 is recommended as a useful marker for differentiating ependymal and other CNS tumors (7). To the best of our knowledge, its utility in SEGA has not been explored yet.

In this study, we investigated CD99 expression in SEGA as an adjunctive marker for diagnostic purposes.

## Method and Materials

Clinical and demographic data of patients were collected. Slides of the five reported SEGA cases were reviewed; the best slide for each one was selected. The tissue sections were deparaffinized, rehydrated, retrieved (to unmask the antigenic epitope) and blocked endogenous peroxidase, incubated with monoclonal antibody (Code: 12E7, Dako, Denmark) to express CD99. Peroxidase labeled polymer (EnVision, Code: K 4061, Dako, Denmark) and substrate-chromogen solution were also used for the IHC staining and counterstained with hematoxylin. Then the slides were reviewed by an expert pathologist. 

Patient consent forms were completed prior to recruitment in our study.

## Case illustration

**Case 1**

A 13-year old girl was admitted with complaint of imbalance and headache. She mentioned deterioration of her vision in the left eye and seizure attacks for two months before admission. Magnetic resonance imaging (MRI) and computed tomography (CT scan) of brain displayed a 4 cm hyper-dense lobulated mass between frontal horns occupying foramen monro, along with subependymal and intraparenchymal calcified nodules in lateral ventricles. The mass was totally removed without any neurological deficit.

**Figure 1:**
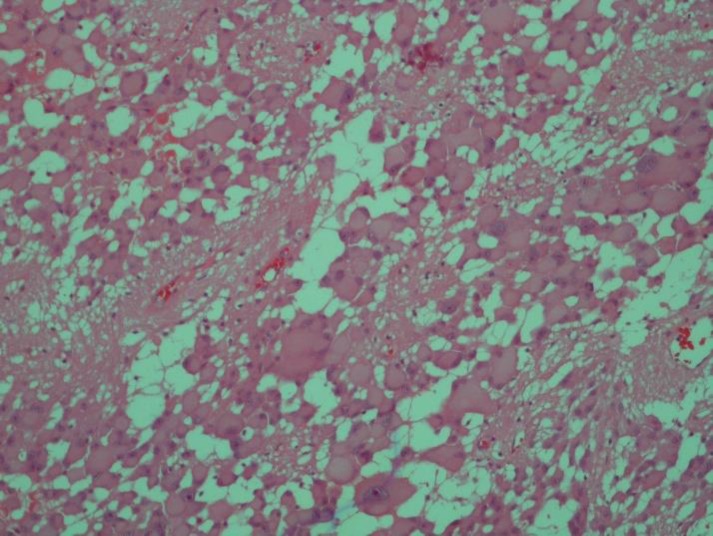
1a. Pleomorphic Multinucleated Tumoral Giant Cells Characteristic of Subependymal Giant Cell Astrocytoma. Hematoxylin –eosine (H&E) staining. Magnification (x400).

**Figure 2:**
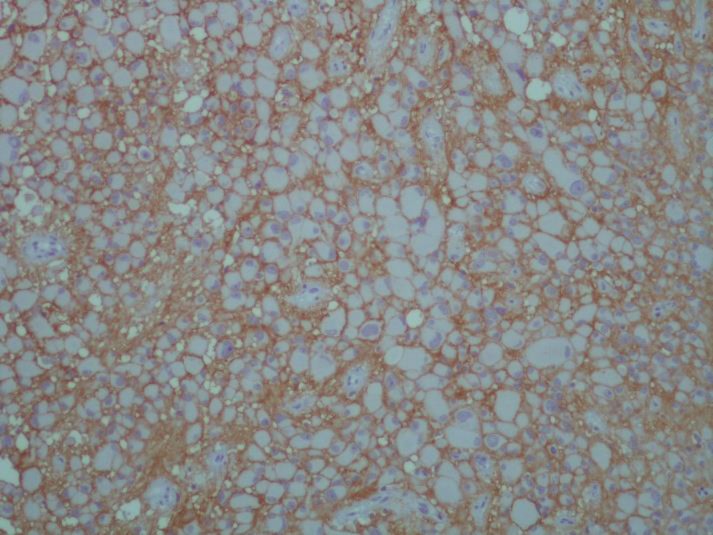
Diffuse Strong Membranous Immunohistochemical Staining of CD99 in Subependymal Giant Cell Astrocytoma. Magnification (x400).

Histopathologic slides revealed hypercellular tumor characterized by large closely packed pleomorphic multinucleated cells with abundant eosinophilic cytoplasm admixed with spindle cells arranged in perivascular arrangements Figure1a. Immunohistochemical staining was performed for GFAP, NSE; additionally, strong positive reaction for CD99 was found in most tumoral cells (Figure 2*)*. After surgery, seizure was controllable with medication and patient discharged without any complication.

**Case 2**

A 56-year old woman presented with severe headache, imbalance and visual loss in emergency ward. She mentioned chronic headache since four months ago. Magnetic resonance imaging revealed a 30x12mm hyper dense lobulated mass in the third ventricle adjacent to foramen monro accompanied by hydrocephaly. Total excision of tumor was performed.

Histopathologic slides showed neoplastic proliferation of huge astrocytic cells with  eccentric nuclei and abundant eosinophilic cytoplasm along with some oval astrocytic cells. Immunohistochemical staining for CD99 and GFAP showed strong membranous immunoreactvity, whereas no reactivity was found for synaptophysin and chromogranin.

The patient discharged without any significant complication. No clinical work up was performed for tuberous sclerosis.

**Case 3**

A 13-year old boy experienced headache and diplopia for one week before admission. On physical exam, papilledema (grade I) was evident. Function of other cranial nerves was intact. His father mentioned history of tuberous sclerosis and seizure when his son was five years old. For controlling seizure, he had consumed Carbamazepine till he encountered severe headache and diplopia. He underwent surgical resection of an obstructive tumor located in the right lateral ventricle. Histopathologic examination showed sheets of large ganglion like cells with eccentric vesicular nuclei, prominent nucleoli and abundant eosinophilic cytoplasm, which was set in fibrillary background. Immunohistochemical staining for GFAP and NSE showed strong reaction of tumoral cells; whereas, few tumoral cells showed positive results for CD99.

**Case 4**

A three-year-old boy was referred to emergency ward with headache, dizziness and blurred vision. On physical examination, no definite finding was observed except visual disturbance. MRI showed an enhanced mass in right anterolateral aspect of foramen monro and in right frontal horn. No clinical work-up for tuberous sclerosis had been performed before.

Histopathologic slides showed proliferated epitheliod gemistocytic cells with vesicular eccentric nuclei and abundant cytoplasm intermixed with spindle cells; immunohistochemical staining showed diffuse reactivity for GFAP, NSE and CD99.

**Case 5**

An 18-year-old boy with a history of Tuberous sclerosis for 10 years was admitted in emergency ward with visual loss and signs of elevated intracranial pressure. On physical exam, multiple skin lesions were evident throughout the body. He consumed carbamazepine and sodium valproate for controlling seizure. MRI showed a large tumoral mass in lateral ventricle. He underwent total excision of the tumor.

Histopathologic slides showed sheets of large gemistocytic like cells with eccentric nuclei and abundant cytoplasm intermixed with spindle and oval cells, which immunohistochemical staining showed diffuse membranous reactivity for CD99. Clinical information of five cases is listed in detail in Table 1.

**Table 1:** Clinical Information of Reported Cases of Sub-ependymal Giant Cell Astrocytoma.

Patient	Age	sex	Clinical presentation	Maximum Tumor size	Brain lesion	Tumor location	Work up/history Tuberous sclerosis	Recurrence	Sub ependymal nodule
1	13	F	Headache & imbalance	4 cm	single	Foramen monro	yes	no	yes
2	56	F	Headache & visual loss	3 cm	single	Third ventricle	no	no	no
3	13	M	Headache, seizure &diplopia	3 cm	single	Right lateral ventricle	Not done	no	no
4	3	M	Seizure& blurred vision	3cm	single	Foramen monro	Not done	no	no
5	18	M	Seizure & visual loss	4.5 cm	single	Lateral ventricle	yes	yes	no

## Discussion

This study attempted to investigate CD99 expression, as a helpful immunohistochemical diagnostic marker in five reported SEGA cases**. **Glial tumors are the most common primary central nervous system neoplasm. Depending on histological tumor type, they can recur or have malignant behavior. According to the World Health Organization (WHO) criteria, glial tumors are divided into various grades and appropriate modality of treatment is performed for each individual (8).

Subependymal giant cell astrocytoma (SEGA), is an indolent tumor, usually occurs in lateral ventricle near foramen monro and often associated with tuberous sclerosis (9). Although multiple studies showed that SEGA is unique in setting of TSC, lack of comprehensive clinical work-up may be a pitfall in its diagnosis (10). Histological feature, with pleomorphic multinucleated eosinophilic giant tumoral cells and elongated oval astrocytes in between, overlaps with other low grade glial tumors, especially gemistocytic astrocytoma, which may be a potential challenge in diagnosis. Although they are usually benign, few cases with frequent mitosis, necrosis and pleomorphism were reported as atypical SEGA, which could really mimic the malignant gliomas, especially glioblastoma. As these tumors are pathologically classified as grade I glioma, differentiating from other gliomas, with similar pathology is crucial, because treatment in SEGA is surgical without any adjunctive therapy.

Multiple ultrastructural and immunohist-ochemical studies have been performed on characteristic nature of tumoral cells in SEGA. Jozwiak et al. (6) concentrated on the morphology of giant cells and demonstrated that neural markers most often expressed are microtubule associated protein (MAP-2), NSE, Somatostatin and Neu. On the other hand, he showed their glial origin with expression of GFAP and S100 protein. Grajkowska et al (11) reported three cases of SEGA with co expression of GFAP, Synapto-physin, S100 and NF. 

 Throughout the literature, CD99, product of MIC2 gene, a transmembrane glycoprotein that is virtually expressed in immature thymocyte and tumors, especially Ewing sarcoma/PNET, is recommended for differentiating ependymal and nonependymal CNS tumors. Mahfouz et al. (12) studied 38 ependymoma and 41 other types of CNS tumors and demonstrated that despite rare weak CD99 expression in few choroid plexus papilloma, CD99 could be a valuable marker for differentiating ependymal tumors and other CNS tumors.

 However, we investigated CD 99 expression in five reported cases of SEGA, and strong membranous immunoreactivity in tumoral giant cells observed in addition to strong reaction for S100, GFAP and NSE. CD99 expression in infiltrating glioma such as gemistocytic astrocytoma is not observed. yet rare types of GBM whereas in those cases neuroimaging and clinical reactivity for CD99 settings could be more helpful. Although multiple studies emphasis that SEGA could be radiologically diagnosed as a serial growing lesion located in caudothalamic groove on the consecutive imaging in background history of tuberous sclerosis (13-15) and pathologic diagnosis should not be challenging, few emerging studies discuss that SEGA could be developed without previous TSC background, so differentiation from other imitators, seems to be indispensable. Prevalence of SEGA is too low, therefore investigating CD99 expression in more tumoral cases is not an easy task.

 Based on our findings, CD99 may be a useful adjunctive marker in differentiating SEGA from other mimickers. 
